# Malaria control along China-Myanmar Border during 2007–2013: an integrated impact evaluation

**DOI:** 10.1186/s40249-016-0171-4

**Published:** 2016-08-10

**Authors:** Jian-Wei Xu, Yong Li, Heng-Lin Yang, Jun Zhang, Zai-Xing Zhang, Ya-Ming Yang, Hong-Ning Zhou, Joshua Havumaki, Hua-Xian Li, Hui Liu, Hua Zhou, Xin-Yu Xie, Jia-Xiang Dong, Yue Zhang, Xiao-Ying Sun, Bo Li, Jia-Yin Li, Yang-Hui Tian, Pi-Yu Wang, Ben-Fu Li

**Affiliations:** 1 Yunnan Institute of Parasitic Diseases, Yunnan Provincial Center of Malaria Research, Yunnan Provincial Collaborative Innovation Center for Public Health and Disease Prevention and Control, Yunnan Provincial Key Laboratory of Vector-borne Diseases Control and Research, Puer, 665000 China; 2 Yunnan Provincial Health and Family Planning Commission, Kunming, 650200 People’s Republic of China; 3 Yunnan Office of Health Poverty Action, Kunming, 650041 People’s Republic of China; 4 World Health Organization Regional Office for the Western Pacific, P.O. Box 2932, 1000 Manila, Philippines; 5 Foundation for Innovative New Diagnostics, 1216 Cointrin, Geneva Switzerland

**Keywords:** Border malaria, Control, Artemisinin resistance, China-Myanmar border

## Abstract

**Background:**

Implementing effective interventions remain a lot of difficulties along all border regions. The emergence of artemisinin resistance of *Plasmodium falciparum* strains in the Greater Mekong Subregion is a matter of great concern. China has effectively controlled cross-border transmission of malaria and artemisinin resistance of *P. falciparum* along the China-Myanmar border.

**Methods:**

A combined quantitative and qualitative study was used to collect data, and then an integrated impact evaluation was conducted to malaria control along the China-Myanmar border during 2007–2013.

**Results:**

The parasite prevalence rate (PPR) in the five special regions of Myanmar was decreased from 13.6 % in March 2008 to 1.5 % in November 2013. Compared with the baseline (PPR in March 2008), the risk ratio was only 0.11 [95 % confidence interval (*CI*), 0.09–0. 14) in November 2013, which is equal to an 89 % reduction in the malaria burden. Annual parasite incidence (API) across 19 Chinese border counties was reduced from 19.6 per 10 000 person-years in 2006 to 0.9 per 10 000 person-years in 2013. Compared with the baseline (API in 2006), the API rate ratio was only 0.05(95 % *CI*, 0.04–0.05) in 2013, which equates to a reduction of the malaria burden by 95.0 %. Meanwhile, the health service system was strengthened and health inequity of marginalized populations reduced along the international border.

**Conclusion:**

The effective collaboration between China, Myanmar and the international non-governmental organization promptly carried out the core interventions through simplified processes. The integrated approaches dramatically decreased malaria burden of Chinese-Myanmar border.

**Electronic supplementary material:**

The online version of this article (doi:10.1186/s40249-016-0171-4) contains supplementary material, which is available to authorized users.

## Multiligual abstracts

Please see Additional file 1 for translations of the abstract into the six official working languanges of the United Nations.

## Background

A population of 3.2 billion is currently at risk for malaria infection on the globe [1]. The Greater Mekong Subregion (GMS) is considered a hot-spot of malaria transmission with around 70 % of the local population at risk of contracting malaria [2, 3]. Within the GMS, China has made extraordinary progress in malaria control [4] and aims to eliminate malaria nationwide by 2020 [5]. On the other hand, Myanmar, which shares a border of 2 185 km with China, is still among the 31 highest burden malaria countries in the world [2, 6]. Importantly, Myanmar exports malaria to its neighboring countries. This seriously impedes progress of malaria elimination in these countries [7]. Additionally, five of the six GMS countries, all except China, have reported artemisinin resistance of *Plasmodium falciparum* [1, 8–12].

Malaria control and elimination together with attempts to contain drug resistance have received much attention along international borders, but effective interventions remain challenging [1, 5, 8]. From 2007 to 2013, intensive efforts have dramatically reduced the number of malaria cases and the parasite prevalence along China- Myanmar border; meanwhile, the drug sensitivity of malaria parasites has not changed significantly [13, 14]. Knowledge and lessons learnt from the border malaria control would benefit the global health. In this context, an impact evaluation was conducted on the cross-border malaria control program.

## Methods

### Study site and population

**Fig. 1:**
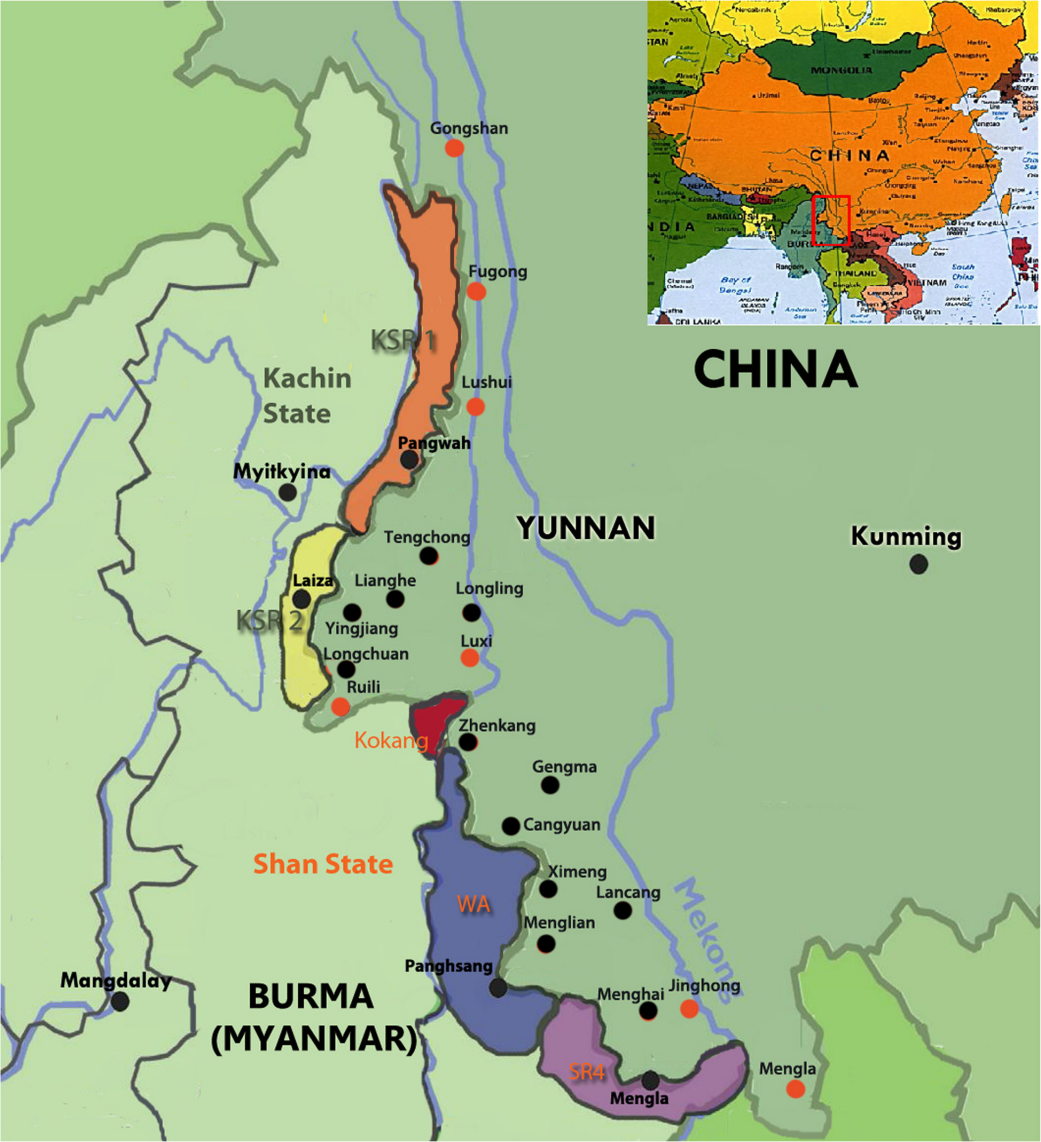
Map of the study site and neighboring region

This study was conducted between January 2008 and May 2014 across 19 border counties of Yunnan Province in China and five special regions in Myanmar (Fig. 1). The study population consisted of 4 687 896 Chinese individuals and 586 000 residents from Myanmar. Notably, there were estimated 110 000 longer term migrants (≥ one month in Myanmar) from China based on the five Special Regions of Myanmar, and 1.5 million short-term migrants from both China and Myanmar who cross the border frequently. More than 50 % of the study population was ethnic minorities [15]. These groups could not usually receive sufficient access to healthcare. Malaria control services of the Myanmar Ministry of Health were unable to effectively cover the five special regions due to ongoing conflicts between the Governments of Union of Myanmar and locally ethnical military.

### Core interventions

The sixth and tenth rounds of the Chinese malaria programs within the Global Fund to fight AIDS, Tuberculosis and Malaria (GFATM) launched in July 2007. The GFATM program established 68 border malaria posts (BMP) at informal border crossing locations throughout the 19 Chinese counties. The BMPs provided diagnosis and treatment for malaria, long lasting insecticidal nets (LLIN) and behavior change communication (BCC) for the 1.5 million short-term migrants. The existing public health facilities were responsible for malaria control of the local residents across 19 border counties in China.

Health Poverty Action (HPA), an UK-based non-government organization (NGO), established 80 malaria diagnoses and treatment stations (MDTS) in the five special regions of Myanmar. The MDTs provided diagnosis and treatment for the residents of Myanmar and the Chinese longer term migrants who were based in the five special regions of Myanmar. In order to address gaps in public sector coverage in Myanmar, the GFATM programs supported community-based services, including, diagnosis and treatment in 102 selected private clinics in communities where MDTS were not available, 475 village malaria workers (VMW) in remote and hyperendemic villages and 30 migrant malaria workers (MMW) in the communities of Chinese longer term migrants. Meanwhile, HPA ran outreach services incorporating LLIN distribution, BCC and support supervision together. The outreach teams trained peer health educators in villages, and supervised operations of MDTS, VMWs and MMWs and gave technical support to them. HPA also worked with the local governments to introduce regulations banning the sale of the fake and sub-standard antimalarial drugs including artemisinin monotherapy.

### Study procedures

Data of process evaluation were collected monthly. In China, the malaria case and its related information such as demographic data and travel history (for identifying the place of infection), were daily collected and reported by “Chinese Information System for Disease Control and Prevention (CISDCP)” [16]. The travel history was used to identify the infection origin in those cross border people. Malaria patients that had once been in Myanmar within one month prior to laboratory diagnosis were defined as imported malaria [5].

In five special regions of Myanmar, HPA’s Health Information System which was established and maintained by the GFATM programs collected and reported the number of malaria cases every month. Meanwhile, cross-sectional surveys were conducted to collect data on parasite prevalence among residents between January and March in 2008, 2009, 2010 and 2011; and between September and November in 2012 and 2013. The study design was fixed as described by Wang et al. [17].

The GFATM program supplied antimalarial drugs. Dihydroartemisinin-piperaquine (DP) is the most commonly used artemisinin- based combination therapy (ACT) for treatment of *P. falciparum* malaria. Chloroquine was commonly used for treatment of *P. vivax* malaria. The sensitivity of *P. falciparum to* DP and the sensitivity of *P. vivax* to chloroquine were *in vivo* monitored [13, 14, 18–20].

From November 2013 to May 2014, qualitative study consisting of focus group discussions (FGD) and semi-structured in-depth interviews (SDI) were undertaken to collect qualitative data for the beneficiary- and community-based evaluation [21–23]. Four FGDs were planned for stakeholders and eight FGDs for villagers, 8–10 participants each group. SDIs were planned to administer to 26 key informants. The issues discussed with participants in FGDs and key informants of SDIs included change of malaria burden; impact on public health, healthcare system and social equity; and finally, challenges and recommendations for malaria control in the border region.

### Statistical analysis

The data of core interventions were aggregated and analyzed every six months. The parasite prevalence rate (PPR) and the annual parasite incidence (API) with 95 % confidence intervals (*CI*) were calculated by Epi Info 7 (Centers for Disease Control and Prevention, Atlanta, GA). Risk ratios (RR) with 95 % *CI* in Myanmar were calculated by using the PPR in March, 2008 as the reference. API rate ratios (IRR) with 95 % *CI* were calculated by taking the API of 2008 in Myanmar and the API of 2006 in China as the reference respectively. Qualitative data analysis was conducted by software TAMS 3.0. The data were encoded based on emerging themes and then a codebook was created. Trends in the data were identified by producing matrices which combined and compared with information collected from the different FGDs and key informants. Finally, quantitative and qualitative results were combined and compared [21–23].

## Results

### Core interventions

Core interventions were successfully targeted residents along the China-Myanmar border. Table 1 indicates the numbers of core interventions, including LLINs distributed, diagnostic tests used, short-term migrants who received piperaquine for preventive treatment, cases treated with quality-assured ACTs and chloroquine- primaquine (CQ-PQ).

**Table 1:** Amount of core interventions across Chinese-Myanmar border from 2008 to 2013

Core interventions	2007	2008	2009	2010	2011	2012	2013	Total
Five special regions of Myanmar (Target populations: a population of 586 000 local residents and about 110 000 Chinese immigrants in Myanmar)								
Numbers of long-lasting insecticidal bed nets distributed	13 866	67 753	25 274	13 943	38 134	84 506	93 501	336 977
Number of febrile patients who received parasite-based diagnosis (RDTs or microscopy)	10 706	116 163	13 0684	103 538	26 431	71 289	121 083	579 894
Number of malaria cases treated with ACTs	3 059	27 254	37 192	25 782	2 084	85 10	11 537	106 908
Number of malaria cases treated with CQ-PQ	–	193	18 339	24 580	51 12	3 731	6 642	53 485
Health education								
Number of community health education sessions organized	–	537	579	637	301	187	651	2 892
Number of attendees of community health education sessions	–	47 560	40 099	42 801	19 218	10 657	28 892	189 227
19 counties of China (Target populations: border-crossing migrants, about 1,500,000 person-times each year)								
Numbers of long-lasting insecticidal nets distributed to migrants	9 486	30 867	10 977	1 6275	2 0042	6 6922	3 1031	1 85600
Number of migrants who received preventive treatment	28 976	37 552	47 594	44 479	27 719	46 740	44 269	277 329
Number of migrant febrile patients who received Parasite-based diagnosis (Microscopy)	41 126	75 495	106 234	91 332	70 040	70 690	67 168	522 085
Number of migrant malaria cases treated with ACTs	808	853	1 644	1 184	546	207	125	5 367
Number of migrant malaria cases treated with CQ-PQ	3 056	7 081	8 699	6 044	2 083	1 084	1 220	2 9267
Number of migrants who received IEC materials	36 649	152 989	88 453	98 295	161 194	108 542	211 316	857 438

*Notes*: *RDT* rapid diagnostic test, *ACT* artemisinin-based combination therapy, *CQ-PQ* chloroquine-primaquine, *IEC* information, education and communication

### Reduced malaria burden in Myanmar

**Fig. 2:**
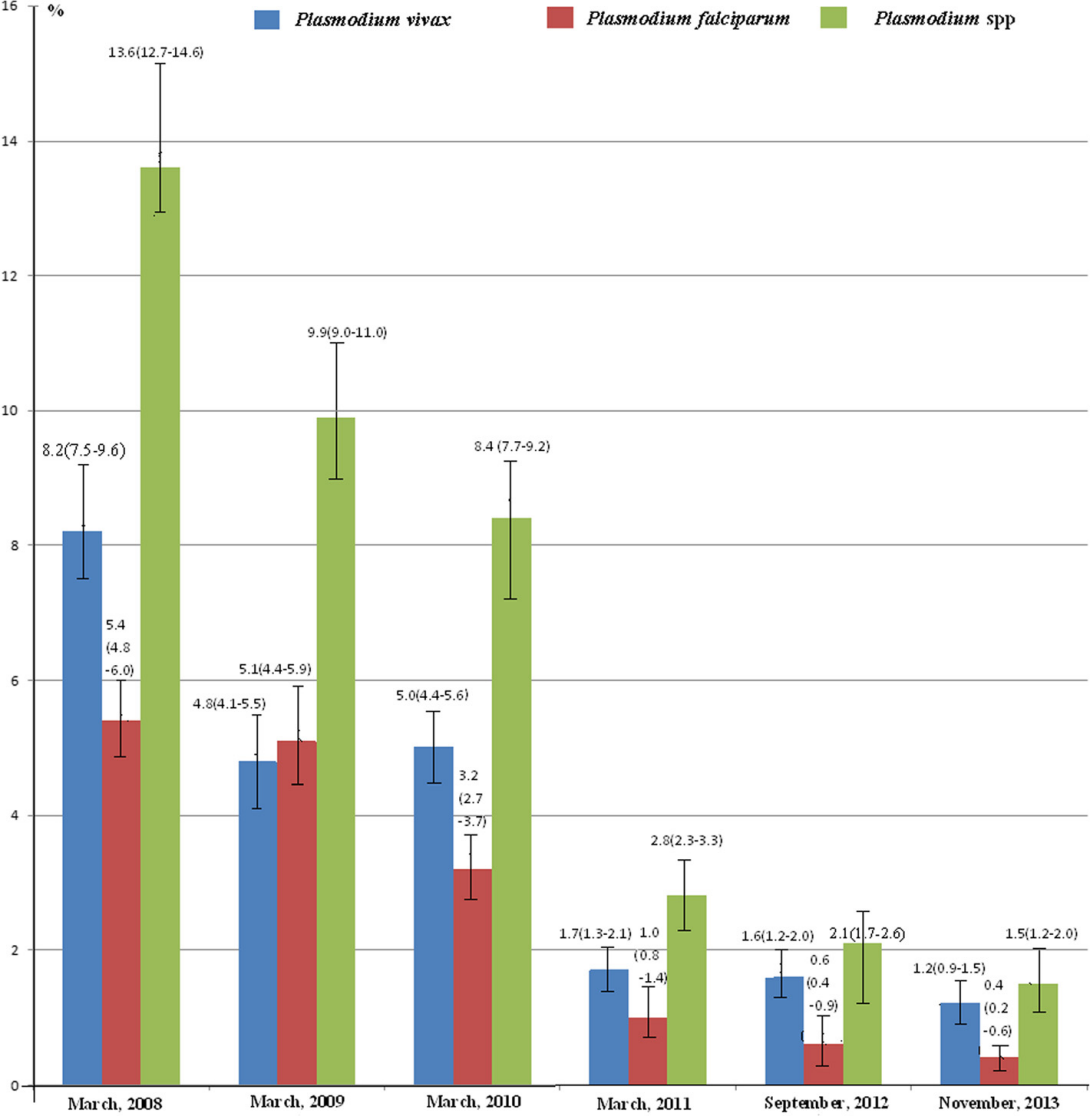
Parasite prevalence rates in the five special regions of Myanmar from March 2008 to November 2013

**Fig. 3:**
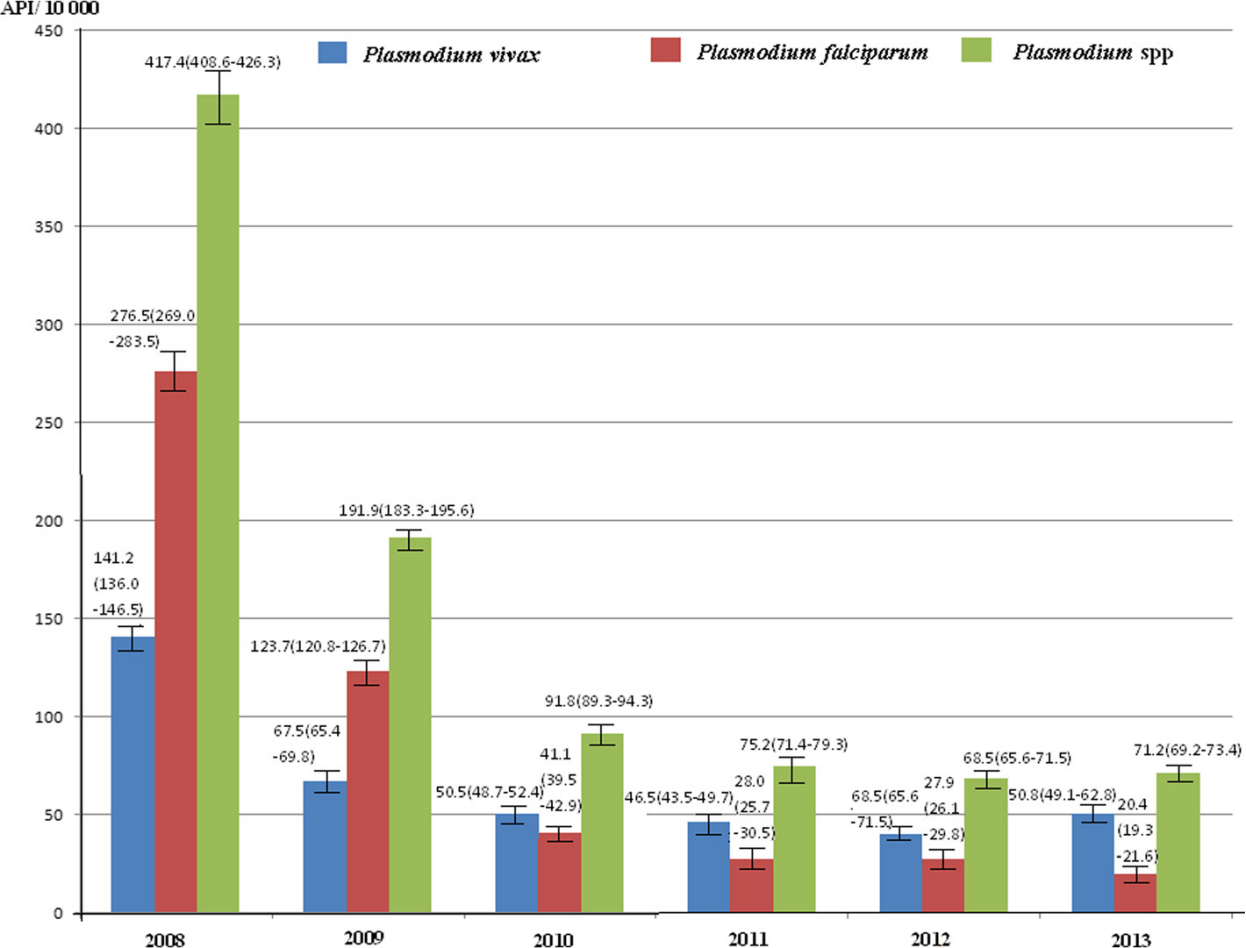
Annual parasite incidences (API) in the five special regions of Myanmar from 2008 to 2013, based on Health Information System of HPA

The malaria burden was gradually reduced since the start of the GFATM interventions in 2008. In Myanmar, the PPR decreased from 13.6 % (95 % *CI*, 12.7–14.6 %) in March, 2008 to 1.5 % (95 % *CI*, 1.2–2.0 %) in November, 2013 (Fig. 2). Compared with the baseline (PPR in March 2008), the RR was only 0.11(95 % *CI*, 0.09–0.14) in November 2013, which is equal to an 89 % reduction in the malaria burden (Table 2). Additionally, the API reduced from 417.4 (95 % *CI*, 408.6–426.3) per 10, 000 person-years in 2008 to 71.2 (95 % *CI*, 69.2–73.4) per 10, 000 person-years in 2013 (Fig. 3). Compared with the API in 2008, API rate ratio (IRR) was only 0.17 (95 % *CI*, 0.16–0. 18) in 2013 (Table 3). There was not any reported malaria-related death during 2008–2013.

**Table 2:** Change of risk ratios in five special regions of Myanmar from 2008 to 2013

Dates surveyed	*Plasmodium vivax*			*Plasmodium falciparum*			*Plasmodium* spp.		
	Number	Risk ratio (95 % *CI*]	*P*- value	Number	Risk ratio (95 % *CI*]	*P*- value	Number	Risk ratio (95 % *CI*]	*P*- value
Jan-Mar, 2008 (Baseline, *n* = 5585)	460	Reference	–	299	Reference	–	761	Reference	–
Jan-Mar, 2009 (*n* = 3600)	172	0.58 (0.49–0.69)	<0.0001	184	0.95 (0.80–1.14)	0.6452	358	0.73 (0.65–0.82)	<0.0001
Jan-Mar, 2010 (*n* = 5090)	255	0.61 (0.52–0.71)	<0.0001	161	0.59 (0.49–0.71)	<0.0001	429	0.62 (0.55–0.69)	<0.0001
Jan-Mar, 2011 (*n* = 4069)	69	0.21 (0.16–0.26)	<0.0001	44	0.20 (0.15–0.28)	<0.0001	113	0.20 (0.17–0.25)	<0.0001
Sep-Nov, 2012 (*n* = 4561)	71	0.19 (0.15–0.24)	<0.0001	27	0.11 (0.07–0.16)	<0.0001	98	0.16 (0.13–0. 19)	<0.0001
Sep-Nov, 2013 (*n* = 4517)	52	0.14 (0.11–0. 19)	<0.0001	18	0.07 (0.05–0. 12)	<0.0001	70	0.11 (0.09–0. 14)	<0.0001

**Table 3:** Change of annual parasite incidence rate ratios based on project reports in five special regions of Myanmar from 2008 to 2013

Years (*n* = person-years^a^]	Vivax malaria			Falciparum malaria			All confirmed malaria		
	Number of cases	Rate ratio (95 % *CI*]	*P-* value	Number of cases	Rate ratio (95 % *CI*]	*P-* value	Number of cases	Rate ratio (95 % *CI*]	*P*- value
2008 (Baseline, *n* = 195 849)	2 765	Reference	–	5 409	Reference	–	8 174	Reference	–
2009 (*n* = 544 876)	3 680	0.48 (0.46–0.50)	<0.0001	6 738	0.45 (0.43–0.46)	<0.0001	10 455	0.46 (0.45–0.47)	<0.0001
2010 (*n* = 554 143)	2 798	0.36 (0.34–0.38)	<0.0001	2 279	0.15 (0.14–0.16)	<0.0001	5 084	0.22 (0.21–0.23)	<0.0001
2011 *(n* = 187 803)	873	0.33 (0.31–0.36)	<0.0001	526	0.10 (0.09–0.11)	<0.0001	1 413	0.18 (0.17–0.19)	<0.0001
2012 (*n* = 308 067)	1 251	0.29 (0.27–0.31)	<0.0001	860	0.10 (0.09–0.11)	<0.0001	2 111	0.16 (0.16–0. 17)	<0.0001
2013 (*n* = 626 125)	3 181	0.36 (0.34–0. 38)	<0.0001	1 279	0.07 (0.07–0. 08)	<0.0001	4 460	0.17 (0.16–0. 18)	<0.0001

^a^The GFATM stopped its Chinese operations between May and December, 2011, HPA’s Health Information System in the five special regions of Myanmar established and maintained by the GFATM grants, could run only for four months (January to April), *n* = 4 X 563409/12 = 187803 person-years. In 2012, HPA spent the first half of the year to recruit new staff and restore project activities, *n* = 6 × 572 678/12 = 286 339 person-years

In Kachin Special Region II (KR2), stakeholders of FGDs still listed malaria as the most important public health problem. However, they said that the number of malaria cases had been reduced dramatically, and that there were no malaria-related deaths after intervention. Participants of internally displaced people in Je Yang Hka and Hpun Lum Yang said that “Malaria was the most life-threatening disease before intervention in KR2. For instance, there were more than 20 deaths of malaria in Ban Dong, a village with approximately 4 000 residents in 2006”.

In eastern Shan Special Region IV (SR4), stakeholders of FGDs described malaria as the most important public health problem before the intervention. However, malaria was just listed as the fourth or fifth one following the intervention. Participants considered malaria as the most burdensome communicable disease. They said that *P*. falciparum malaria prevalence and mortality were very high before the intervention, and currently, morbidity was very low. The deputy director of the health department of SR4 said that “the proportion of malaria was about 40–60 % of outpatients before 2006”. The chief of the Health Bureau of Nan Bang County of SR4 said that “many people died of malaria prior to the intervention, and then various people left Nan Bang because of malaria fearfulness. However, malaria is uncommon now; there is only one or two imported malaria cases from other areas of Myanmar”. Participants of Man Beng Long and Man Beng Mai villages said that: “Malaria was the most common disease prior to the intervention, 40 %–50 % of people contracting malaria each year. Some families had three or four malaria patients a year. However, malaria was currently scarce. It was difficult to find a malaria patient among 100 people. Only men who ever worked in the forest, such as lumbering workers, could contract malaria”.

In Shan Special Region II (locally called Wa State), the director of the health department said that “many intervention activities had been carried out in Wa State. The malaria prevalence was very low after these interventions”. The project manager of HPA of Wa State said that: “About twenty percent of febrile patients were malaria prior to the intervention. In 2005, more than 300 malaria cases were confirmed by Rapid diagnosis test (RDT) during three work days in Lian He Township. However, it was difficult to find malaria cases at all there following the intervention”.

### Eliminating malaria in china

**Fig. 4:**
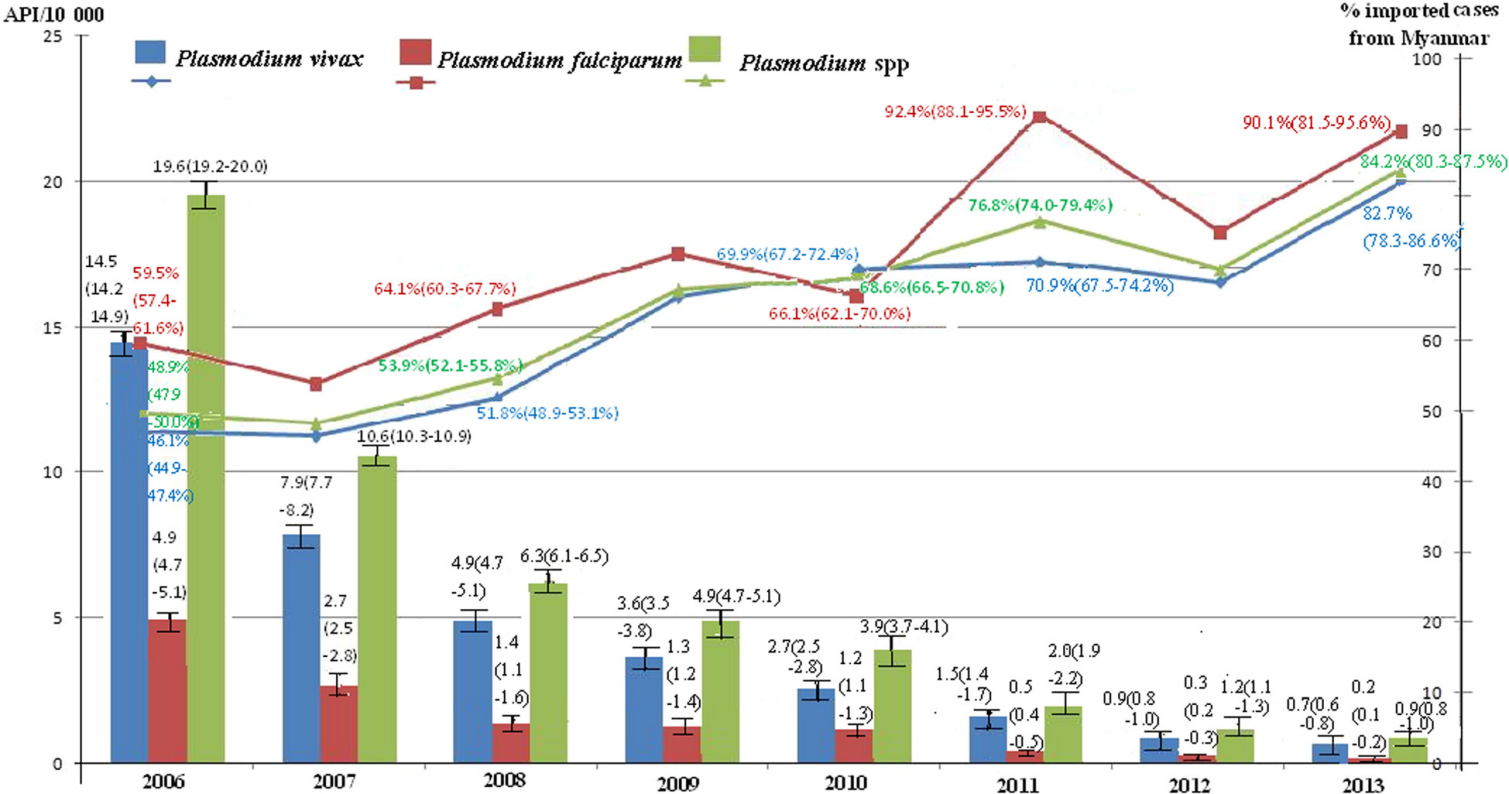
Annual parasite incidences (API) and the proportions of imported malaria cases in 19 counties of China, from 2006 to 2013, based on Chinese information system for disease control and prevention

Most people of 19 border counties in China were at a high risk of malaria infection prior to the interventions. The API was 19.6 (95 % *CI*, 19.2–20.0) per 10 000 person-years in 2006, and then the API remarkably declined to 0.9 (95 % *CI*, 0.8–1.0) per 10 000 person-years in 2013 (Fig. 4). From 2006 to 2013, the number of imported malaria cases of Yunnan Province of China from Myanmar was 4 340, 2 291, 1 566, 1 524, 1 258, 734, 388, and 356, respectively. The proportion of imported cases in total malaria cases increased from 48.9 % (95 % *CI*, 47.9–50.0 %) in 2006 to 84.2 % (95 % *CI*, 80.3–87.5 %) in 2013 (Fig. 4). Compared to the baseline (API in 2006), the IRR was only 0.05 (95 % *CI*, 0.04–0.05) in 2013 (Table 4), which equates to a reduction of the malaria burden by 95.0 %. These indicate major progress towards elimination.

**Table 4:** Change of annual parasite incidence rate ratios based on Chinese information system for disease control and prevention across 19 counties of Yunnan Province, China, from 2006 to 2013

Years (*n* = person-years]	Vivax malaria			Falciparum malaria			All confirmed malaria		
	Number of cases	Rate ratio (95 % *CI*]	*P*- value	Number of cases	Rate ratio (95 % *CI*]	*P*- value	Number of cases	Rate ratio (95 % *CI*]	*P*- value
2006(Baseline, *n* = 4 521 402)	6 570	Reference	–	2 197	Reference	–	8 874	Reference	–
2007 (*n* = 4 562 688)	3625	0.55 (0.53–0.57)	<0.0001	1 209	0.55 (0.51–0.58)	<0.0001	4 847	0.54 (0.52–0.56)	<0.0001
2008 (*n* = 4 603 659)	2235	0.33 (0.32–0.35)	<0.0001	665	0.30 (0.27–0.32)	<0.0001	2 903	0.32 (0.31–0.34)	<0.0001
2009 (*n* = 4 644 719)	1686	0.25 (0.24–0.26)	<0.0001	595	0.26 (0.24–0.29)	<0.0001	2 283	0.25 (0.24–0.26)	<0.0001
2010 (*n* = 4 687 896)	1261	0.19 (0.17–0.20)	<0.0001	570	0.25 (0.23–0.27)	<0.0001	1 833	0.20 (0.19–0.21)	<0.0001
2011 (*n* = 4 729 147)	732	0.11 (0.10–0.11)	<0.0001	223	0.10 (0.08–0.11)	<0.0001	956	0.10 (0.10–0.11)	<0.0001
2012 (*n* = 4 770 122)	417	0.06 (0.05–0.07)	<0.0001	137	0.06 (0.05–0.07)	<0.0001	556	0.06 (0.05–0.06)	<0.0001
2013 (*n* = 4 800 944)	342	0.05 (0.04–0.05)	<0.0001	81	0.03 (0.03–0.04)	<0.0001	423	0.05 (0.04–0.05)	<0.0001

The director of the Health Bureau of Mang Shi City, the former headmaster of Zhe Fang Middle School said that “The project had made impressive progress in reducing the malaria burden. Malaria transmission among school children has been a major problem prior to the intervention; but malaria was scarcely following the implementation of the GFATM programs”.

Malaria was accounted for 40–50 % of total communicable diseases prior to the intervention. Stakeholders in both Mengla County and Jinghong City listed malaria as the most important public health problem before the GFATM program. Notably, they had difficulty to rank malaria at all after the intervention. Stakeholders in Mengla County said that “Malaria was no longer a public health problem because there was only one or two locally infected malaria cases each year in this county with a population of 310 000”. However, stakeholders in Jinghong City said that “Malaria was still a major public health problem despite the fact that there were only from three to five imported malaria cases each year. Existing malaria vectors, cross-border migration of population and imported malaria from neighboring countries may reestablish malaria transmission again”.

As results of surveillance of drug sensitivity, six hundred and three patients with *Plasmodium vivax* infections treated with chloroquine were completed a 28 day follow-up. The fever clearance time (FCT) and asexual parasite clearance times (APCT) were, respectively, 22.2 ± 10.2 and 38.1 ± 12.6 h. A total of 594 (98.5 %, 95 % *CI*, 97.2–99.3 %) were adequate clinical and parasitological response (ACPR), and only nine (1.5 %, 95 % *CI* 0.7–2.8 %) patients were late clinical failure (LCF). The proportions of ACPR did not change significantly from 2008 to 2013 (*X*^2^ = 0.86, *P* = 0.6496) [13]. Two hundred and forty three *P*. falciparum malaria patients treated with DP were completed a 42 day follow-up. The FCT and APCT were, respectively, 36.5 ± 10.9 and 43.5 ± 11.8 h, and there was an increasing trend of both FCT (*F* = 268.41, *P* < 0.0001) and APCT (*F* = 88.6, *P* < 0.0001) from 2007 to 2013. Eight (3.3 %, 95 % *CI*, 1.4–6.4 %) patients present parasitaemias on day three after medication; however they were spontaneous cure at day four without further treatment. In total, two hundred and forty one of 243 (99.2 %; 95 % *CI*, 97.1 - 99.9 %) patients were ACPR. The proportions of ACPR did not change substantially from 2007 to 2013 (*X*^2^ = 2.81, *P* = 0.7288) [14].

### Impact to health service system

In China, the services of malaria diagnosis, treatment, and prevention for those who frequently travel across the China-Myanmar border were strengthened. In the five special regions of Myanmar, GFATM ensured that all residents had access to free quality-assured primary health care within two hours walking distance from their homes.

GFATM-supported training enhanced capacity of HPA staff, locally governmental health officers in Myanmar, and CDC staff in China on malaria control and project management. Four hundred and fifty-six received training in diagnoses including microscopy and RDT, and administering proper treatment. One thousand and forty community-based workers including VMWs, MMWs and private practitioners received training in using RDT for diagnoses and dosing for malaria treatment. One thousand two hundred and ninety-eight community peer educators were trained to provide health education. Traditional birth attendants (TBA) were also trained to distribute LLINs and give consultation on malaria for pregnant women.

The interventions made a positive impact on basic health services. In China, an employee of Dan Zhu Border Malaria Post said that “the program had improved the capacity of the village clinic”. In SR4 of Myanmar, both the HPA manager and the director of the health bureau said that “The local government had recruited and trained health care workers prior to the GFATM projects. However, in a case without GFATM grants, loss of health workers would collapse the local health service system”.

### Reduced social inequality

Communities including migrants and ethnical minorities along the China-Myanmar border are one of the vulnerable groups in the region. As a fact, populations who are living in malaria endemic areas are largely poor and marginalized, and at a higher risk of malaria infection. These core interventions improved accessibility and quality of malaria control services for them. Healthcare was offered regardless of gender. The VMWs and MMWs ensured communities in remote and highly endemic areas access to the free diagnosis and treatment. The poorest and the most vulnerable populations were given priority to receive LLINs with an additional one given to a pregnant woman through TBAs. Women were encouraged to actively participate in community health education and social mobilization, development of materials of information, education and communication (IEC) and strategies of BCC. The results of a household survey in KSR2 conducted between June and September of 2013, indicated that the bed net to person ratio reached 1:1.96 (i.e., more than one net for every two people), and the LLIN to person ratio reached 1:2.52. People in the poorest families were much more likely to sleep under ITNs/LLINs than those in richer families [23]. These showed the positive impact of these interventions in reduction of social inequality.

## Discussion

The China-Myanmar border region was once among the highest burden regions of malaria in the world. In 2003, a malaria epidemic took place in Kokang Region, Myanmar. One thousand three hundred and ninety-two new cases of malaria and 125 deaths were detected across 30 villages in three districts during 13 days,from the 7^th^ to 19^th^, November [24]. In December 2007, an active case detection was performed in Wa State, 453 febrile patients were found and tested by microscopy. The slide positivity rate was 60 % (270/453) and 90.74 % (245/270) of parasites were *P. falciparum* [25]. In October 2009, the parasite prevalence rate of 23.3 % (36/159) was detected in school children and the *P. falciparum* prevalence rate of was 14.5 % (23/159) in KR1 [26].

The five special regions of Myanmar are one of the major imported malaria sources in China. This situation seriously impedes malaria elimination in China [24–27]. The reduced parasite prevalence rate and API showed that the core interventions of the GFATM programs decreased malaria burden by 89 % between 2008 and 2013 in Myanmar (Fig. 2, Tables 2 and 3); and by 95 % between 2006 and 2013 in China (Fig. 3, Table 4). The number of imported malaria cases had decreased by 92 %, from 4 340 in 2006 to 356 in 2013 in Yunnan Province of China. These efforts have promoted malaria elimination in China.

*P. falciparum* resistance to artemisinin has been confirmed in the GMS, all countries except China [1], and threatens control and elimination of malaria worldwide [10, 28]. Artemisinin resistance is rampant in Myanmar. The country is considered the main route through which drug-resistant *P. falciparum* will spread from Southeast Asia to the Indian Subcontinent and then on to Africa [29, 30]. In the China-Myanmar border areas, artemisinin was highly efficacious for the treatment of falciparum malaria by 2013 [14, 31, 32] and molecular markers of artemisinin resistance was not found by 2012 [33, 34]. These results indicate that these core interventions have prevented the development and spread of artemisinin resistance.

Huang et al. reported kelch13 propeller domain mutations in *P. falciparum* that was sampled from China-Myanmar border during 2009–2012 [35], however, the K13-propeller mutation was not detected in *P. falciparum* that was sampled from 2013 (unpublished data) to2014 [36]. The relative prevalence of *P. falciparum* parasites carrying K13-propeller mutations was investigated in 55 sites of ten administrative regions in Myanmar. K13-propeller mutation was discovered in most parts of Myanmar except China-Myanmar border [29]. Population size and genetic diversity of malaria parasites, and multi-clonal infections are correlated to the spread of anti-malarial resistance as well as transmission intensity [37]. Increasing access to quality-assured diagnosis and treatment with ACTs can limit drug resistance, and can contribute to outcome improvement in patients [38]. Most of these populations are ‘hill tribes’ on the China-Myanmar border. Poor access to quality-assured diagnosis and treatment gave rise to self-treatment and delayed standard treatment [22, 39]. The results of a cross-sectional study showed that only 32.0 % of febrile patients sought treatment within 24 h, while 79.6 % of febrile patients purchased anti-malarial drugs themselves from the retail sector before the core interventions of the GFATM programs [22]. Free quality-assured diagnosis and treatment with ACTs to *P. falciparum* and the introduction of regulations successfully beat the chaotic anti-malarial market in the five special regions of Myanmar. BCC activities improved knowledge and awareness about seeking qualified treatment, and compliance with the standard treatment regimen. These core interventions successfully prevented drug resistance.

The China-Myanmar border is extremely porous. Chinese official border crossing stations recorded 10 999 677 person-times of border crossings in 2009. Political isolation has resulted in a lack of governmental health service structure in the five special regions of Myanmar [17]. BMPs in China and MDTS integrated with private and community-based facilities in Myanmar dramatically improved malaria diagnosis and treatment service.

Myanmar staffs received training in China through the project. This dramatically improved local and migrant access to quality-assured health service. BMPs and MDTS only provided malaria diagnoses and treatment before July, 2009. Patients who did not have malaria were not in position to receive any treatment of BMPs and MDTS. This led to fewer and fewer febrile patient visiting BMPs and MDTS. To ensure these facilities working effectively, BMPs and MDTS were expanded into public health facilities. The improvement in primary healthcare attracted febrile patients to visit these facilities.

Malaria Control along the international border can easily be affected by the political climate. In this project, an international NGO, HPA, implemented core interventions in the five Special Regions of Myanmar with financial support from GFATM and technical assistance from China under an agreement between China and Myanmar. This simplified the process and ensured carrying out these prompt and efficacious core interventions by avoiding complexity required for diplomatic coordination.

The study presents several limitations too. First, the evaluation was unable to address potential confounding, e. g. socioeconomic development, climate change and deforestation. However, China-Myanmar border was ever a high malaria transmission area [1, 2]. These intensive control efforts played a dominant role in the change of the malaria situation. Second, Global Fund R6 and R10 were control projects. The requirement of covering as many areas as possible did not permit to use control groups. This leads to difficult to clearly link the declined API to each special intervention. Third, there were no appropriate governmental health service structures prior to the GFATM projects in the five Special Regions of Myanmar. HPA could only carry out some of these core interventions in parts of target areas because of a lack of trained staff and logistics in the first two project years. Some data such as the number of cases treat with CQ-PQ were unavailable in 2007, and only 193 cases of *P. vivax* malaria were treated in 2008. Fourth, the epidemiological data were sparse in five Special Regions of Myanmar prior to the intervention. Available data were mainly collected by the HPA’s Health Information System. This leads to the number of malaria cases detected was fewer in 2008 than in 2009 because of the fact that the project did not completely cover all project areas in Myanmar in 2008. The system could only run for four months (January-April) because the GFATM abruptly stopped its China operations for eight months in 2011. HPA spent the first half of the year to recruit new staff and restored project activities, the project could only operate for six months (July to December) in 2012 (Table 3). API was also calculated as an indicator for evaluation in spite of this situation. A cross-sectional survey of parasite prevalence was undertaken to compensate for the limitation every year. Fifth, the cross-sectional survey of parasite prevalence was conducted from January to March when malaria transmission was lower during 2008–2011, and from September to November when malaria transmission was higher during 2012–2013 (Table 2). However, if anything, this time limitation of the cross-sectional surveys may just underestimate the impact of core interventions on malaria burden.

## Conclusions

In conclusion, integrated approaches reduced about 90 % of malaria burden on the China-Myanmar border. The intervention contributed to malaria elimination in China and containment of artemisinin resistance. Meanwhile, it strengthened the healthcare system and reduced social inequity of marginalized populations. However, further regional efforts and funding for cross-border interventions are still needed to achieve malaria elimination along the international border.

## Abbreviations

ACPR, Adequate clinical and parasitological response; ACT, Artemisinin- based combination therapy; APCT, Asexual parasite clearance times; API, Annual parasite incidence; BCC, Behavior change communication; BMP, Border malaria posts; CDC, Center for disease control and prevention; CI, Confidence interval; CISDCP: Chinese Information System for Disease Control and Prevention; CQ-PQ, Chloroquine- primaquine; DP, Dihydroartemisinin-piperaquine; FCT, Fever clearance time; FGD, Focus group discussion; GFATM, The Global Fund to fight AIDS, Tuberculosis and Malaria; GMS, Greater Mekong Subregion; HPA, Health Poverty Action; IEC, Information, education and communication; IRR, Annual parasite incidence rate ratio; ITN, insecticide-treated net; K13, Kelch gene on chromosome 13; KR2, Kachin Special Region II,Myanmar; LCF, Late clinical failure; LLIN, long lasting insecticidal net; MDTS, Malaria diagnoses and treatment station; MMW, Migrant malaria worker; NGO, Non-government organization; PPR, Parasite prevalence rate; RDT, Rapid diagnosis test; RR, Risk ratio; SDI, Semi-structured in-depth interviews; SR4, Shan Special Region IV,Myanmar; TBA, Traditional birth attendant; VMW, Village malaria worker.

## Additional files

Additional file 1: Multilingual abstracts in the six official working languages of the United Nations. (PDF 757 kb) Additional file 2: Figure S1. Map of project location relative to neighboring countries. Five special regions in Myanmar = Kachin Special Region I (KSR1), Kachin Special Region II (KSR2), Kokang, Shan Special Region II (Wa) and Shan Special Region IV (SR4). 12 counties of Round 6 in China (black dots) = Tengchong(TC), longchuan(LC), Yingjiang(YJ), Lianghe (LH), Longling(LL), Zhenkang(ZK), Gengma(GM),Cangyuan(CY), Ximeng(XM), Menglian(ML), Lancang(LC) and Menghai(MH). Seven counties of Round 10 in China (red dots) = Gongshan(GS), Fugong(FG), Lushui(LS), Ruili(RL), Luxi, Jinghong(JH) and Mengla(MLa). **Table S1.** Parasite prevalence in five special regions of Myanmar from 2008 to 2013. **Table S2.** Annual parasite incidence (API/10,000) based on project reports in five special regions of Myanmar from 2008 to 2013. **Table S3.** Annual parasite incidence (API/10, 000) and number of imported malaria cases based on China information system for disease control and prevention (CISDCP) in 19 counties, China from 2006 to 2013. (DOC 472 kb)
