# Supplementary material for: Malaria control along China-Myanmar Border during 2007–2013: an integrated impact evaluation
Source: Infect Dis Poverty. 2016 Aug 10;5:75. doi: 10.1186/s40249-016-0171-4 (PMC4979141; doi:10.1186/s40249-016-0171-4)
Supplement: Additional file 1: — Multilingual abstracts in the six official working languages of the United Nations. (PDF 757 kb) [file 40249_2016_171_MOESM1_ESM.pdf]

Translation of the abstract into the six official working languages of the United Nations

السيطرة على الملاريا على طول الحدود الفاصلة بين الصين وميانمار في 2013-2007: تقييم متكامل للأثر

جيان-وي كسو، يونغ لي، هينغ-لين يانغ، جون زانغ، زاي-كسنغ زانغ، يا-مِنغ يانغ، هونغ-نِنغ زاو، جوشوا هافوماكي، هوا-كسيان لي، هوي ليو، هوا زاو، كسن-يو كسي، جيا-كسيانغ دونغ، يو زانغ، كسياو-يِنغ سَن، بو لي، جيا-يِن لي، يانغ-هوي تيان، بي-يو وانغ، بين-فو لي

خلاصة
خلفية: تبقى عملية اجراء تدخلات ناجعة تلاقي صعوبات كثيرة في جميع المناطق الحدودية. ويعدّ طروء سلالات المتصورات المنجلية المقاومة لمادة ارتيميسينين في مقاطعة ميكونغ الكبرى أمراً باعثاً على القلق. لقد سيطرت الصين بفاعلية على انتقال الملاريا عبر الحدود وكذلك على عدوى المتصورات المنجلية المقاومة للارتيسيمينين على طول الحدود بين الصين وميانمار.
الطرائق: تم استخدام دراسة كمّية نوعية لجمع البيانات، وتمّ اجراء تقييم متكامل لأثر عملية السيطرة على الملاريا على طول الحدود بين الصين وميانمار للفترة 2013-2007.
النتائج: قلّ معدل انتشار الطفيلي (ppr) في المناطق الخمس الخاصة في ميانمار من 13.6% في آذار 2008 الى 1.5% في تشرين الثاني 2013. وبالمقارنة مع الخط الأساس (معدل انتشار الطفيلي في 2008)، كانت نسبة الخطر 0.11 [95% مهلة زمنية للثقة (*cl*)، 0.14-0.09) في تشرين الثاني 2013، وهو ما يساوي انخفاضا بنسبة 89% للإصابة بالملاريا. وقد تم تقليل ورود الطفيلي السنوي (API) عبر 19 مقاطعة حدودية صينية من 19.6 لكل 10,000 شخص عام 2006 الى 0.9 لكل 10,000 شخص عام 2013. وبالمقارنة مع الخط الأساس (معدل ورود الطفيلي السنوي في 2006)، فإن نسبة معدل الورود كانت فقط 0.05 (95% cl، 0.05-0.04) عام 2013، والذي يعادل انخفاضا بنسبة 95.0% للإصابة بالملاريا. في تلك الأثناء، فقد تم تعزيز نظام الخدمة الصحية وتقليل التعسف الصحي للفئات المهمشة على طول الحدود الدولية.
الاستنتاجات: إنّ التعاون الفعال بين الصين، وميانمار، والمنظمات غير الحكومية العالمية أدى بصورة حثيثة التدخلات الرئيسية عن طريق عمليات يسيرة. ولقد قللت المنهجيات المتكاملة عبء الاصابة بالملاريا بصورة كبيرة على حدود الصين وميانمار.

Translated from English version into Arabic by Muther J. Alohmayed, through

TRANSLATORS
WITHOUT BORDERS

中缅边境 2007-2013 年疟疾控制：综合性影响评估

Jian-Wei Xu, Yong Li, Heng-Lin Yang, Jun Zhang, Zai-Xing Zhang, Ya-Ming Yang, Hong-Ning Zhou, Joshua Havumaki, Hua-Xian Li, Hui Liu, Hua Zhou, Xin-Yu Xie, Jia-Xiang Dong, Yue Zhang, Xiao-Ying Sun, Bo Li, Jia-Yin Li, Yang-Hui Tian , Pi-Yu Wang, Ben-Fu Li

摘要
引言: 在边境地区开展有效防治干预，总有许多困难。恶性疟原虫在大湄公河次区域出现了青蒿素抗性株，引起了很大关注。中国在中缅边境有效地控制了疟疾跨境传播和恶性疟原虫的青蒿素抗性。
方法: 使用定量和定性研究相集合的方法收集数据和资料，对中缅边境 2007-2013 年疟疾控制开展综合性影响评估。
结果: 缅甸五个特区居民疟原虫带虫率(PPR), 从 2008 年 3 月的 13.6% 下降到 2013 年 11 月的 1.5%，与基线（2008 年 3 月 PPR）相比, 2013 年 11 月相对带虫率仅 0.11(95%可信区间, 0.09 - 0. 14)，相当于疟疾负担减少 89%。中国 19 个边境县的年带虫发病率（API），从 2006 年的 19.6/万下降到 2013 年的 0.9/万，与基线（2006 年的 API）相比, 2013 年相对发病率仅为 0.05 (95%可信区间, 0.04 - 0..05)，相当于疟疾负担减少 95%。同时，加强了边境地区的卫生服务系统，减少了边缘化人群健康之不公平性。
结论: 通过中国、缅甸和国际非政府组织的有效合作，采用简化途径，及时有效地实施了各项核心防治干预措施。这些综合性措施极大地降低了中缅边境的疟疾负担。

Translated from English version into Chinese by Xu Jian-wei

La lutte contre le paludisme le long de la frontière sino-birmane entre 2007 et 2013: évaluation intégrée des impacts

Jian-Wei Xu, Yong Li, Heng-Lin Yang, Jun Zhang, Zai-Xing Zhang, Ya-Ming Yang, Hong-Ning Zhou, Joshua Havumaki, Hua-Xian Li, Hui Liu, Hua Zhou, Xin-Yu Xie, Jia-Xiang Dong, Yue Zhang, Xiao-Ying Sun, Bo Li, Jia-Yin Li, Yang-Hui Tian , Pi-Yu Wang, Ben-Fu Li

Résumé
Contexte: La mise en œuvre d’interventions efficaces reste très difficile dans les régions frontalières. L’émergence d’une résistance à l’artémisinine chez des souches de *Plasmodium falciparum* dans la sous-région du Grand Mékong est une source d’inquiétude majeure. La Chine a contrôlé efficacement la transmission transnationale du paludisme et de la résistance à l’artémisinine de *P. falciparum* le long de sa frontière avec le Myanmar (Birmanie).

Méthodes: Une étude quantitative et qualitative a été réalisée dans le but de récolter des données, puis une évaluation des impacts intégrée a été menée sur le contrôle du paludisme le long de la frontière sino-birmane entre 2007 et 2013.
Résultats: Le taux de prévalence parasitaire (PPR) dans les cinq régions spéciales du Myanmar ont diminué de 13,6 % en mars 2008 à 1,5 % en novembre 2013. Par rapport à la valeur à la date de référence (PPR en mars 2008), le facteur de risque était de seulement 0,11 (intervalle de confiance [IC] à 95 % 0,09-0,14] en novembre 2013, ce qui équivaut à 89 % de diminution de la charge paludique. L'incidence annuelle parasitaire (API) dans les 19 pays frontaliers de la Chine a été réduite de 19,6 pour 10 000 personnes-an en 2006 à 0,9 pour 10 000 en 2013. Par rapport à la valeur à la date de référence (API en 2006) elle était de seulement 0,05 (IC à 95 % 0,04-0,05) en 2013, ce qui équivaut à une réduction de la charge paludique de 95,0 %. Pendant ce temps, le système de soins s'est renforcé et les inégalités de santé des populations marginalisées se sont réduite le long de la frontière internationale.
Conclusion: La collaboration efficace entre la Chine, le Myanmar et des organisations non gouvernementales internationales a permis un déploiement rapide des interventions essentielles en simplifiant les procédures. Ces approches intégrées ont considérablement diminué la charge paludique à la frontière entre la Chine et le Myanmar.

Translated from English version into French by Suzanne Assenat, through

TRANSLATORS
WITHOUT BORDERS

Мероприятия по борьбе с малярией вдоль границы Китая и Мьянмы в течение 2007-2013 годов: оценка комплексного воздействия

Жиан-Вэй Ксу, Ёнг Лий, Генг-Лин Янг, Юн Жанг, Заи-Ксинг Жанг, Я-Минг Янг, Гонг-Нинг Жоу, Джошуа Хавумаки, Гуа-Ксян Лий, Гуи Лю, Гуа Жоу, Ксин-Ю Кси, Жиа-Ксианг Донг, Ю Жанг, Ксяо-Инг Сун, Бо Лий, Жя-Ин Лий, Янг-Гуи Тян , Пи-Ю Ванг, Бен-Фу Лий
(Jian-Wei Xu, Yong Li, Heng-Lin Yang, Jun Zhang, Zai-Xing Zhang, Ya-Ming Yang, Hong-Ning Zhou, Joshua Havumaki, Hua-Xian Li, Hui Liu, Hua Zhou, Xin-Yu Xie, Jia-Xiang Dong, Yue Zhang, Xiao-Ying Sun, Bo Li, Jia-Yin Li, Yang-Hui Tian , Pi-Yu Wang, Ben-Fu Li)

Резюме
Исходная информация: Осуществление эффективного вмешательства остается трудным во всех приграничных районах. Появление в субрегионе Большого Меконга штаммов *Plasmodium falciparum,* резистентных к артемизинину, вызывает большую озабоченность. Китай осуществляет эффективный контроль трансграничного распространения малярии и резистентного к артемизинину вида малярии *P. Falciparum* вдоль границы Китая и Мьянмы.
Методы: Для сбора данных использовались результаты совокупного количественного и качественного исследования; затем проведена оценка комплексного воздействия мероприятий по борьбе с малярией вдоль границы Китая и Мьянмы в течение 2007-2013 годов.
Результаты: Показатель распространенности паразита (PPR) в пяти особых районах Мьянмы снизился с 13,6% в марте 2008 года до 1,5% в ноябре 2013 года. По сравнению с исходным показателем (PPR в марте 2008 года) коэффициент риска составил в ноябре 2013 года лишь 0,11% [доверительный интервал 95% (*CI*); 0,09-0,14], что соответствует снижению уровня заболевания малярией на 89%. Годовой показатель заболеваемости (API) в 19 приграничных уездах Китая снизился с 19,6 на 10 тысяч человеко-лет в 2006 году до 0,9 на 10 тысяч человеко-лет в 2013 году. По сравнению с исходным показателем (API в 2006 г.) соотношение показателей API составило в 2013 году лишь 0,05 (доверительный интервал 95% (*CI*); 0,04-0,05), что соответствует снижению уровня заболевания малярией на 95%. Одновременно, в приграничных районах международной границы была укреплена система медицинского обслуживания и сократилось неравенство в области охраны здоровья маргинализированных групп населения.
Вывод: В результате эффективного сотрудничества между Китаем, Мьянмой и международной неправительственной организацией с помощью упрощенных процедур оперативно осуществлены основные мероприятия. Комплексный подход существенно сократил уровень заболевания малярией в приграничных районах Китая и Мьянмы.

Translated from English version into Russian by Tatyana Johnson, through

TRANSLATORS
WITHOUT BORDERS

Control de la malaria a lo largo de la frontera de China con Myanmar durante el período 2007 – 2013: evaluación integrada del impacto

Jian-Wei Xu, Yong Li, Heng-Lin Yang, Jun Zhang, Zai-Xing Zhang, Ya-Ming Yang, Hong-Ning Zhou, Joshua Havumaki, Hua-Xian Li, Hui Liu, Hua Zhou, Xin-Yu Xie, Jia-Xiang Dong, Yue Zhang, Xiao-Ying Sun, Bo Li, Jia-Yin Li, Yang-Hui Tian, Pi-Yu Wang, Ben-Fu Li

Resumen

Antecedentes: Sigue habiendo muchas dificultades para instrumentar intervenciones de manera eficaz en todas las regiones fronterizas. Es motivo de gran preocupación la aparición de ciertas cepas de *Plasmodium falciparum* resistentes a la artemisina en la subregión del Mekong y sus zonas circundantes. China ha logrado controlar la transmisión de la malaria allende frontera y la resistencia del *P. falciparum* a la artemisina a lo largo de su frontera con Myanmar.

Métodos: Se empleó un estudio combinado cuantitativo y cualitativo para reunir datos, y luego se realizó una evaluación integrada del impacto en el control de la malaria a lo largo de la frontera de China con Myanmar durante el período 2007-2013.

Resultados: En las cinco regiones especiales de Myanmar, se redujo el índice de prevalencia del parásito (PPR por su sigla en inglés) del 13,6% en marzo de 2008 al 1,5% en noviembre de 2013. Con respecto a la línea de base (PPR de marzo de 2008), el índice de riesgo fue de solo 0,11 [95% de intervalo de confianza (*CI*), 0,09-0,14] en noviembre de 2013, lo cual equivale a una reducción del 89% en la incidencia de la malaria. Se redujo la incidencia anual del parásito (API) en 19 condados fronterizos de 19,6 por 10.000 personas por año en 2006 a 0,9 por 10.000 personas por año en 2013. Con respecto a la línea de base (API de 2006), el ratio del índice de API fue de solo 0,05 (95% *CI*, 0.04-0.05) en 2013, lo cual equivale a una reducción de la incidencia de la malaria del 95,0%. Entretanto, se fortaleció el sistema de atención médica y se redujo la inequidad en la atención a las poblaciones desprotegidas de las zonas fronterizas.

Conclusión: La eficaz cooperación de China, Myanmar y la organización no gubernamental internacional permitió llevar a cabo, sin demora, las intervenciones fundamentales por medio de procesos simplificados. Los enfoques integrados permitieron reducir radicalmente la incidencia de la malaria a lo largo de la frontera de China con Myanmar.

Translated from English version into Spanish by Mónica Algazi, through

TRANSLATORS
WITHOUT BORDERS
