# Supplementary material for: Malaria control along China-Myanmar Border during 2007–2013: an integrated impact evaluation
Source: Infect Dis Poverty. 2016 Aug 10;5:75. doi: 10.1186/s40249-016-0171-4 (PMC4979141; doi:10.1186/s40249-016-0171-4)
Supplement: Additional file 2: Figure S1. — Map of project location relative to neighboring countries. Five special regions in Myanmar = Kachin Special Region I (KSR1), Kachin Special Region II (KSR2), Kokang, Shan Special Region II (Wa) and Shan Special Region IV (SR4). 12 counties of Round 6 in China (black dots) = Tengchong(TC), longchuan(LC), Yingjiang(YJ), Lianghe (LH), Longling(LL), Zhenkang(ZK), Gengma(GM),Cangyuan(CY), Ximeng(XM), Menglian(ML), Lancang(LC) and Menghai(MH). Seven counties of Round 10 in China (red dots) = Gongshan(GS), Fugong(FG), Lushui(LS), Ruili(RL), Luxi, Jinghong(JH) and Mengla(MLa). Table S1. Parasite prevalence in five special regions of Myanmar from 2008 to 2013. Table S2. Annual parasite incidence (API/10,000) based on project reports in five special regions of Myanmar from 2008 to 2013. Table S3. Annual parasite incidence (API/10, 000) and number of imported malaria cases based on China information system for disease control and prevention (CISDCP) in 19 counties, China from 2006 to 2013. (DOC 472 kb) [file 40249_2016_171_MOESM2_ESM.doc]

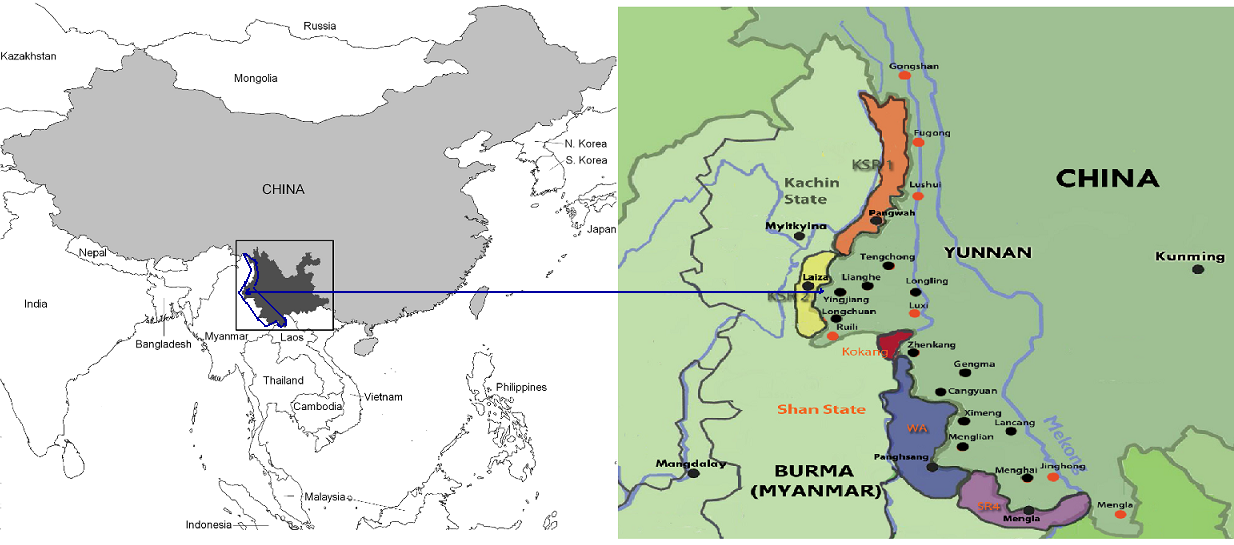


**Figure 1 Map of project location relative to neighboring countries**

**Five special regions in Myanmar** = Kachin Special Region I (KSR1), Kachin Special Region II (KSR2), Kokang, Shan Special Region II (Wa) and Shan Special Region IV (SR4). **12 counties of Round 6 in China (black dots)** = Tengchong(TC), longchuan(LC), Yingjiang(YJ), Lianghe (LH), Longling(LL), Zhenkang(ZK), Gengma(GM)，Cangyuan(CY), Ximeng(XM), Menglian(ML), Lancang(LC) and Menghai(MH). **Seven counties of Round 10 in China (red dots)** = Gongshan(GS), Fugong(FG), Lushui(LS), Ruili(RL), Luxi, Jinghong(JH) and Mengla(MLa).

**Table 1 Parasite prevalence in five special regions of Myanmar from 2008 to 2013**

| **Areas and years (n=Number surveyed)** | **Plasmodium vivax** | | | **Plasmodium falciparum** | | | **Plasmodium spp (including others)** | | |
| --- | --- | --- | --- | --- | --- | --- | --- | --- | --- |
|  | **Number (%, 95%CI)** | **Risk ratio (95%CI)** | **P- value** | **Number (%, 95%CI)** | **Risk ratio (95%CI)** | **P- value** | **Number (%, 95%CI)** | **Risk ratio (95%CI)** | **P- value** |
| March, 2008（n=5585） | 460 (8.2, 7.5-9.0) | 1 | - | 299 (5.4, 4.8-6.0) | 1 | - | 761 (13.6, 12.7-14.6) | 1 | - |
| March, 2009  （n= 3600） | 172 (4.8, 4.1-5.5) | 0.58 (0.49-0.69) | < 0.0001 | 184 (5.1, 4.4-5.9) | 0.95 (0.80-1.14) | 0.6452 | 358 (9.9, 9.0-11.0) | 0.73 (0.65-0.82) | < 0.0001 |
| March, 2010（n=5090） | 255 (5.0, 4.4-5.6) | 0.61 (0.52-0.71) | < 0.0001 | 161 (3.2, 2.7-3.7) | 0.59 (0.49-0.71) | < 0.0001 | 429 (8.4, 7.7-9.2) | 0.62 (0.55-0.69) | < 0.0001 |
| March, 2011 （n= 4069） | 69 (1.7, 1.3-2.1) | 0.21 (0.16-0.26) | < 0.0001 | 44 (1.0, 0.8-1.4) | 0.20 (0.15-0.28) | < 0.0001 | 113 (2.8, 2.3-3.3) | 0.20 (0.17-0.25) | < 0.0001 |
| September, 2012（n=4561） | 71 (1.6, 1.2-2.0) | 0.19 (0.15-0.24) | < 0.0001 | 27 (0.6, 0.4-0.9) | 0.11 (0.07-0.16) | < 0.0001 | 98 (2.1, 1.7-2.6) | 0.16 (0.13-0. 19) | < 0.0001 |
| November, 2013（n=4517 ） | 52 (1.2, 0.9-1.5) | 0.14 (0.11-0. 19) | < 0.0001 | 18 (0.4, 0.2-0.6) | 0.07 (0.05-0. 12) | < 0.0001 | 70 (1.5, 1.2-2.0) | 0.11 (0.09-0. 14) | < 0.0001 |

**Table 2 Annual parasite incidence (API/10,000) based on project reports in five special regions of Myanmar from 2008 to 2013**

| **Areas and years (n=person-years)** | **Plasmodium vivax** | | | **Plasmodium falciparum** | | | **Plasmodium spp (including others)** | | |
| --- | --- | --- | --- | --- | --- | --- | --- | --- | --- |
|  | **Number ( API, 95%CI)** | **Rate ratio ( 5%CI)** | **P- value** | **Number (API, 95%CI)** | **Rate ratio (5%CI)** | **P- value** | **Number (API, 95%CI)** | **Rate ratio ( 5%CI)** | **P- value** |
| 2008 (n=195849） | 2765 (141.2, 136.0-146.5) | 1 | - | 5409 (276.2, 269.0-283.5) | 1 | - | 8174 (417.4, 408.6-426.3) | 1 | - |
| 2009 (n=544876） | 3680 (67.5, 65.4-69.8) | 0.48 (0.46-0.50) | < 0.0001 | 6738 (123.7, 120.8-126.7) | 0.45 (0.43-0.46) | < 0.0001 | 10455 (191.9, 188.3-195.6) | 0.46 (0.45-0.47) | < 0.0001 |
| 2010 (n=554143） | 2798 (50.5, 48.7-52.4) | 0.36 (0.34-0.38) | < 0.0001 | 2279 (41.1, 39.5-42.9) | 0.15 (0.14-0.16) | < 0.0001 | 5084 (91.8, 89.3-94.3) | 0.22 (0.21-0.23) | < 0.0001 |
| 2011（n=187803） | 873 (46.5, 43.5-49.7) | 0.33 (0.31-0.36) | < 0.0001 | 526 (28.0, 25.7-30.5) | 0.10 (0.09-0.11) | < 0.0001 | 1413 (75.2, 71.4-79.3) | 0.18 (0.17-0.19) | < 0.0001 |
| 2012（n=308067） | 1251 (40.6, 38.4-42.9) | 0.29 (0.27-0.31) | < 0.0001 | 860 (27.9, 26.1-29.8) | 0.10 (0.09-0.11) | < 0.0001 | 2111 (68.5, 65.6-71.5) | 0.16 (0.16-0. 17) | < 0.0001 |
| 2013（n=626125） | 3181 (50.8, 49.1-52.6) | 0.36 (0.34-0. 38) | < 0.0001 | 1279 (20.4, 19.3-21.6) | 0.07 (0.07-0. 08) | < 0.0001 | 4460 (71.2, 69.2-73.4) | 0.17 (0.16-0. 18) | < 0.0001 |

**Table 3 Annual parasite incidence (API/10, 000) and number of imported malaria cases based on China information system for disease control and prevention (****CISDCP) in 19 counties, China from 2006 to 2013**

| **Areas and years (n= person-years)** | **Plasmodium vivax** | | | **Plasmodium falciparum** | | | **Plasmodium spp (including others)** | | | **Number and proportion imported** | | |
| --- | --- | --- | --- | --- | --- | --- | --- | --- | --- | --- | --- | --- |
|  | **Number ( API, 95%CI)** | **Rate ratio ( 5%CI)** | **P- value** | **Number (API, 95%CI)** | **Rate ratio (5%CI)** | **P- value** | **Number (API, 95%CI)** | **Rate ratio ( 5%CI)** | **P- value** | **Vivax (%, 95%CI)** | **Falciparum (%, 95%CI)** | **Total (%, 95%CI)** |
| 2006（n=4521402） | 6570(14.5, 14.2-14.9) | 1 | - | 2197(4.9, 4.7-5.1) | 1 | - | 8874(19.6, 19.2-20.0) | 1 | - | 3032(46.1, 44.9-47.4) | 1308(59.5, 57.4-61.6) | 4340(48.9, 47.9-50.0) |
| 2007 (n=4562688） | 3625(7.9, 7.7-8.2) | 0.55, (0.53-0.57) | <0.0001 | 1209(2.7, 2.5-2.8) | 0.55, (0.51-0.58) | <0.0001 | 4847(10.6 10.3-10.9) | 0.54, (0.52-0.56) | <0.0001 | 1649(45.5, 43.9-47.1) | 642(53.1, 50.2-55.9) | 2291(47.3, 45.9-48.7) |
| 2008（n=4603659） | 2235(4.9, 4.7-5.1) | 0.33, (0.32-0.35) | <0.0001 | 665(1.4, 1.3-1.6) | 0.30, (0.27-0.32) | <0.0001 | 2903(6.3, 6.1-6.5) | 0.32, (0.31-0.34) | <0.0001 | 1140(51.0, 48.9-53.1) | 426(64.1, 60.3-67.7) | 1566(53.9, 52.1-55.8) |
| 2009 (n=4644719） | 1686(3.6, 3.5-3.8) | 0.25, (0.24-0.26) | <0.0001 | 595(1.3, 1.2-1.4) | 0.26, (0.24-0.29) | <0.0001 | 2283(4.9, 4.7-5.1) | 0.25, (0.24-0.26) | <0.0001 | 1095(65.9, 62.6-67.2) | 429(72.1, 68.2-75.7) | 1524(66.8, 64.8-68.6) |
| 2010（n=4687896） | 1261(2.7, 2.5-2.8) | 0.19, (0.17-0.20) | <0.0001 | 570(1.2, 1.1-1.3) | 0.25, (0.23-0.27) | <0.0001 | 1833(3.9, 3.7-4.1) | 0.20, (0.19-0.21) | <0.0001 | 881(69.9, 67.2-72.4) | 377(66.1, 62.1-70.0) | 1258(68.6, 66.5-70.8) |
| 2011（n=4729147） | 732(1.5, 1.4-1.7) | 0.11, (0.10-0.11) | <0.0001 | 223(0.5, 0.4-0.5) | 0.10, (0.08-0.11) | <0.0001 | 956(2.0, 1.9-2.2) | 0.10, (0.10-0.11) | <0.0001 | 519(70.9, 67.5-74.2) | 206(92.4, 88.1-95.5) | 734(76.8, 74.0-79.4) |
| 2012（n=4770122） | 417(0.9, 0.8-1.0) | 0.06, (0.05-0.07) | <0.0001 | 137(0.3, 0.2-0.3) | 0.06, (0.05-0.07) | <0.0001 | 556(1.2, 1.1-1.3) | 0.06, (0.05-0.06) | <0.0001 | 283(67.9, 63.1-72.3) | 103(75.2, 67.1-82.2) | 388(69.8, 65.8-73.6) |
| 2013（n=4800944） | 342(0.7, 0.6-0.8) | 0.05, (0.04-0.05) | <0.0001 | 81(0.2, 0.1-0.2) | 0.03, (0.03-0.04) | <0.0001 | 423(0.9, 0.8-1.0) | 0.05, (0.04-0.05) | <0.0001 | 283(82.7, 78.3-86.6) | 73(90.1, 81.5-95.6) | 356(84.2, 80.3-87.5) |
|  |  |  |  |  |  |  |  |  |  | v | f | t |
